# Urban agrobiodiversity, health and city climate adaptation plans

**DOI:** 10.2471/BLT.22.288857

**Published:** 2022-12-01

**Authors:** Mary C Sheehan

**Affiliations:** a Health Policy and Management Department, Johns Hopkins Bloomberg School of Public Health, 615 North Wolfe St, Baltimore, Maryland, 21205 United States of America.

## Abstract

**Objective:**

To identify the scope and nature of agricultural biodiversity actions within the climate adaptation plans of a sample of large world cities.

**Methods:**

I evaluated data from the 2021 Cities Climate Adaptation Actions database curated by the Carbon Disclosure Project. Cities with a population over 1 million and reporting at least one adaptation action were included. I identified actions involving agriculture and biodiversity using a framework consisting of five agrobiodiversity categories: urban and peri-urban land use and water management, and urban food supply chains, food availability and food environments. I also identified reported health co-benefits and health sector involvement.

**Findings:**

Of 141 cities reviewed, 61 cities reported actions on agricultural biodiversity, mostly supporting land use or water management. Key health outcomes addressed were illnesses linked to air pollution and excessive heat and vector-borne diseases, corresponding with cities’ major health concerns. Greenhouse gas mitigation was also addressed by many cities. Fewer cities reported actions in food categories or concern for noncommunicable diseases or poor nutrition. Nearly two thirds of cities (40/61) reported health co-benefits or health-sector involvement for at least one intervention. A higher proportion of the 43 cities in low- and middle-income countries reported agrobiodiversity actions and health co-benefits than the 18 cities in high-income countries.

**Conclusion:**

Cities are key partners in achieving sustainable global agriculture that promotes health and supports climate and biodiversity goals. Cities can enhance this role through climate adaptation plans with strong health engagement, a focus on nature-based solutions and greater emphasis on food and nutrition.

## Résumé

**Objectif:**

Définir la nature et l'étendue des actions de biodiversité agricole dans le cadre des plans d'adaptation au climat sur un échantillon de grandes villes du monde.

**Méthodes:**

J'ai examiné les informations provenant de la base de données du rapport 2021 sur les actions des villes en matière d'adaptation au climat, publié par l'organisation Carbon Disclosure Project. J'ai retenu les villes comptant plus d'un million d'habitants et ayant adopté au moins une mesure d'adaptation. J'ai ensuite repéré les mesures impliquant l'agriculture et la biodiversité en utilisant un cadre constitué de cinq catégories d'agrobiodiversité: utilisation urbaine et périurbaine des terres, gestion des eaux, chaînes d'approvisionnement alimentaire urbain, disponibilité de la nourriture et environnements alimentaires. Enfin, j'ai identifié les avantages connexes observés en matière de santé, mais aussi la participation du secteur de la santé.

**Résultats:**

Sur 141 villes étudiées, 61 ont mentionné des actions liées à la biodiversité agricole, surtout dans les domaines de l'utilisation des terres ou de la gestion des eaux. En termes de santé, les principaux problèmes évoqués étaient des pathologies liées à la pollution de l'air et à la chaleur extrême ainsi que des maladies à transmission vectorielle, ce qui correspond aux problèmes sanitaires majeurs rencontrés dans les villes. De nombreuses agglomérations ont également cité la réduction des émissions de gaz à effet de serre. Elles étaient en revanche moins nombreuses à avoir entrepris des actions dans des catégories liées à la nourriture, ou à exprimer une inquiétude quant aux maladies non transmissibles ou à une mauvaise alimentation. Près de deux tiers des villes (40/61) ont constaté des avantages connexes en matière de santé ou une participation du secteur pour au moins une intervention. Les 43 villes situées dans des pays à revenu faible et intermédiaire ont davantage fait état de mesures liées à l'agrobiodiversité et d'avantages sanitaires connexes que les 18 villes situées dans les pays à revenu élevé.

**Conclusion:**

Les villes sont des partenaires clés dans la mise en œuvre d'une agriculture mondiale durable qui privilégie la santé et contribue à la réalisation des objectifs relatifs à la biodiversité et au climat. Elles peuvent renforcer ce rôle grâce à des plans d'adaptation au climat qui montrent un engagement fort en faveur de la santé, se concentrent sur les solutions inspirées de la nature et accordent une plus grande attention à l'alimentation et à la nutrition.

## Resumen

**Objetivo:**

Identificar el alcance y la naturaleza de las medidas de biodiversidad agrícola en los planes de adaptación al clima de una muestra de grandes ciudades del mundo.

**Métodos:**

Se evaluaron los datos de la base de datos 2021 Cities Climate Adaptation Actions, elaborada por el Carbon Disclosure Project. Se incluyeron las ciudades con una población superior a un millón de habitantes y que informaron de al menos una medida de adaptación. Se identificaron las medidas relacionadas con la agricultura y la biodiversidad mediante un marco compuesto por cinco categorías de agrobiodiversidad: uso del suelo urbano y periurbano y gestión del agua, y cadenas urbanas de suministro de alimentos, disponibilidad de alimentos y entornos alimentarios. Asimismo, se identificaron los beneficios colaterales para la salud y la participación del sector sanitario.

**Resultados:**

De las 141 ciudades analizadas, 61 informaron de medidas sobre la biodiversidad agrícola, en su mayoría de apoyo al uso del suelo o a la gestión del agua. Los principales resultados en materia de salud que se abordaron fueron las enfermedades relacionadas con la contaminación del aire y el calor excesivo y las enfermedades transmitidas por vectores, que se corresponden con las principales preocupaciones sanitarias de las ciudades. Además, muchas ciudades abordaron la mitigación de los gases de efecto invernadero. Hubo menos ciudades que informaron de medidas en las categorías de alimentos o de la preocupación por las enfermedades no transmisibles o la mala nutrición. Casi dos tercios de las ciudades (40/61) informaron de beneficios colaterales para la salud o de la participación del sector sanitario en al menos una intervención. Un mayor porcentaje de las 43 ciudades de países de ingresos bajos y medios informaron de medidas relacionadas con la agrobiodiversidad y los beneficios colaterales para la salud que las 18 ciudades de países de ingresos altos.

**Conclusión:**

Las ciudades son asociadas clave para lograr una agricultura global sostenible que promueva la salud y apoye los objetivos climáticos y de biodiversidad. Las ciudades pueden potenciar esta función a través de planes de adaptación al clima con un fuerte compromiso sanitario, un enfoque en soluciones basadas en la naturaleza y un mayor énfasis en la alimentación y la nutrición.

## ملخص

**الغرض:**

تحديد نطاق وطبيعة إجراءات التنوع البيولوجي الزراعي ضمن خطط التكيف مع المناخ لعينة من مدن العالم الكبيرة.

**الطريقة:**

لقد قمت بتقييم البيانات من قاعدة بيانات إجراءات التكيف مع المناخ في المدن لعام 2021، والتي يرعاها مشروع الكشف عن الكربون. تم تضمين المدن التي يزيد عدد سكانها عن 1 مليون نسمة، والتي أبلغت عن إجراء تكيف واحد على الأقل. لقد حددت الإجراءات التي تشمل الزراعة والتنوع البيولوجي باستخدام إطار عمل يتكون من خمس فئات للتنوع البيولوجي الزراعي: استخدام الأراضي الحضرية وشبه الحضرية وإدارة المياه، وسلاسل إمدادات الغذاء الحضرية، وتوافر الغذاء والبيئات الغذائية. كما حددت أيضًا الفوائد الصحية المشتركة المُبلغ عنها، ومشاركة القطاع الصحي.

**النتائج من:**

بين 141 مدينة تمت مراجعتها، أبلغت 61 مدينة عن إجراءات تتعلق بالتنوع البيولوجي الزراعي، يدعم أغلبها استخدام الأراضي أو إدارة المياه. كانت النتائج الصحية الرئيسية التي تم تناولها هي الأمراض المرتبطة بالتلوث الهوائي، والحرارة المفرطة، والأمراض المنقولة، والتي تتوافق مع المخاوف الصحية الرئيسية للمدن. وكان موضوع التخفيف من غازات الاحتباس الحراري قد تم تناوله بواسطة العديد من المدن. أبلغ عدد أقل من المدن عن اتخاذ إجراءات في فئات الأغذية، أو القلق بشأن الأمراض غير المعدية، أو سوء التغذية. أبلغ ثلثا المدن تقريباً (40/61)عن منافع صحية مشتركة، أو مشاركة في القطاع الصحي في تدخل واحد على الأقل. أبلغت نسبة أعلى من الـ 43 مدينة في الدول ذات الدخل المنخفض والدخل المتوسطة عن إجراءات التنوع البيولوجي الزراعي، والفوائد الصحية المشتركة مقارنة بالمدن في 18 دولة ذات دخل مرتفع.

**الاستنتاج:**

المدن هي الشركاء الرئيسيون في تحقيق الزراعة العالمية المستدامة، التي تعزز الصحة وتدعم أهداف المناخ والتنوع البيولوجي. يمكن للمدن أن تعزز هذا الدور من خلال خطط التكيف مع المناخ مع مشاركة صحية قوية، والتركيز على الحلول القائمة على الطبيعة، وزيادة التأكيد على الغذاء والتغذية.

## 摘要

**目的:**

旨在确定示例性世界大城市气候适应计划项下农业生物多样性行动的范围和性质。

**方法:**

我评估了基于碳披露项目创建的 2021 城市气候适应行动数据库中的数据。人口超过 100 万且报告采取了至少一项适应行动的城市均被纳入了统计范围。我使用由五个农业生物多样性类别构成的框架确定了农业和生物多样性相关行动：城市和城郊土地利用和水资源管理，以及城市食物供应链、食物可供应性和食物环境。我还确认了报告的健康协同效益及卫生部门参与情况。

**结果:**

在审查的 141 个城市中，有 61 个城市报告采取了农业生物多样性相关行动，主要用于支持土地利用或水资源管理。讨论了因空气污染、过热和虫媒病所致疾病的关键健康结果，这与城市的主要健康问题相一致。许多城市还讨论了温室气体减排问题。少数城市报告了针对食物类别所采取的行动或者非传染性疾病或营养不良相关问题。近三分之二的城市 (40/61) 报告了至少一项干预措施的健康协同效益或卫生部门参与情况。在中低收入国家的 43 个城市报告了农业生物多样性行动和健康协同效益，其所占比例高于高收入国家的 18 个城市。

**结论:**

城市是实现全球可持续农业发展、促进健康以及支持气候和生物多样性目标的关键合作伙伴。城市可通过实施气候适应计划充分发挥其作用，包括大力参与健康行动、优先考虑基于自然的解决方案以及提高对食物和营养的重视。

## Резюме

**Цель:**

Определить масштабы и характер мероприятий по сохранению биоразнообразия в сельском хозяйстве в соответствии с планами по адаптации к климатическим изменениям в выборке крупных городов мира.

**Методы:**

Автор оценил данные из базы данных Cities Climate Adaptation Actions за 2021 год, подготовленной в рамках проекта по раскрытию информации о выбросах углерода. В выборку были включены города с населением более 1 миллиона человек, сообщившие хотя бы об одном мероприятии по адаптации. Автор определил действия в области сельского хозяйства и биоразнообразия, используя структуру, состоящую из пяти категорий агробиоразнообразия: управление городским и пригородным землепользованием и водными ресурсами, городские системы поставок продуктов питания, наличие продовольствия и продовольственная среда. Он также определил сопутствующие преимущества для здоровья и участие сектора здравоохранения.

**Результаты:**

Из 141 рассмотренного города о мероприятиях по биоразнообразию в сельском хозяйстве сообщил 61 город, в основном это были действия, связанные с поддержкой землепользования или управлением водными ресурсами. К основным последствиям для здоровья были отнесены заболевания, связанные с загрязнением воздуха и перегревом, а также трансмиссивные заболевания, что соответствует основным проблемам здравоохранения в городах. Во многих городах также рассматривались вопросы о снижении выбросов парниковых газов. В меньшем количестве городов сообщалось о действиях в категориях продуктов питания или о беспокойстве по поводу неинфекционных заболеваний или неудовлетворительного питания. О сопутствующих преимуществах для здоровья или об участии сектора здравоохранения по крайней мере в одном мероприятии сообщили около двух третей городов (40/61). О мероприятиях по сохранению агробиоразнообразия и сопутствующих преимуществах для здоровья сообщила большая часть из 43 городов в странах с низким и средним уровнем дохода в сравнении с 18 городами в странах с высоким уровнем дохода.

**Вывод:**

Города являются ключевыми партнерами в достижении устойчивости мирового сельского хозяйства, способствующей укреплению здоровья и достижению целей в области климата и биоразнообразия. Города могут способствовать усилению этой роли с помощью планов по адаптации к климатическим изменениям при активном участии системы здравоохранения, уделяя особое внимание природосберегающим решениям и более пристальное внимание продовольствию и питанию.

## Introduction

The twin crises of climate change and biodiversity loss and their interaction with the agriculture and food system create a complex web of impacts on human and planetary health.^1^^–^^4^ Climate change can harm health in direct ways such as illness from excessive heat, and indirect ways such as losses due to extreme weather that lead to mental illness.^2^^–^^4^ The changing climate is also altering the stability of ecosystems in which humans and other species have thrived for millennia.^5^ Human health depends on ecosystems with abundant diversity of species for filtering clean air and fresh water, and maintaining productive soils. Deterioration of this diversity can lead to imbalanced ecosystems, resulting in infectious diseases, food insecurity and poor nutrition.^6^^,^^7^ The agricultural and food system is a driver of both climate change and biodiversity loss. Food production is also a major determinant of health since undernutrition in a context of poverty leaves 820 million people hungry, while excess nutrition leaves 2 billion people overweight or obese and at risk of noncommunicable diseases.^8^

To raise awareness of the interconnected nature of ecosystems and human health, the World Health Organization and the Convention on Biological Diversity, in 2015, drew up a strategic framework providing guidance on sustainable healthy agriculture, food production and nutrition.^9^ The initiative builds on other long-term work to sustainably improve agriculture and human nutrition by the Consortium of International Agricultural Research Centers and partners such as Bioversity, as well as the Food and Agriculture Organization (FAO) of the United Nations and others. The initiative also links to other international frameworks, including the Paris Climate Agreement, the Sendai Disaster Risk Reduction Framework and the Agenda for Sustainable Development Goals.^10^ In 2019, the EAT-*Lancet* Commission defined strategic goals for a sustainable food system that link to agricultural biodiversity: (i) shifting to healthy diets; (ii) reorienting agriculture towards healthy food production; (iii) sustainable intensification of food production with reduced greenhouse gas emissions; (iv) governance of land and ocean for biodiversity; and (v) halving food loss and waste.^8^

Urban areas are at the heart of these interacting threats and opportunities. Cities are home to over half the world’s population, and are responsible for about 70% of greenhouse gas emissions.^11^ While only a small share of food globally is produced in cities, up to 70% is consumed there.^12^ Agriculture-related deforestation to meet the food needs of urban populations contributes to both greenhouse gas emissions and destruction of the habitat supporting native species.^8^ Expansion of urban areas is also a driver of biodiversity loss.^13^ Meanwhile, sufficient contact with nature is so essential to a healthy environment in cities^14^ that it has been called a public health investment.^15^ These features are particularly notable in the world’s largest cities. Food consumption in the most populous cities generates an important share of global greenhouse gas emissions,^16^ in part due to the intensive transport needs of long food supply chains.^17^ Urban green spaces that are rich in biodiversity more often benefit wealthier areas in large cities.^18^^,^^19^ The informal settlements of some large low- and middle-income country cities also experience more extreme weather-related mortality, morbidity and displacement.^20^

One way in which cities are addressing agricultural biodiversity is through climate adaptation planning. These adaptation goals often overlap with the sustainable development goals (SDGs), particularly in low- and middle-income country cities, most notably those targeting ending poverty (SDG 1); good health and well-being (SDG 3); clean water and sanitation (SDG 6); sustainable cities and communities (SDG 11); and climate action (SDG 13).^21^ A common adaptation strategy is nature-based solutions, which aim to “leverage the power of healthy ecosystems to protect people.”^22^ Such strategies are supported by a growing evidence base and address multiple climate hazards simultaneously. For example, enhancing urban vegetation, such as extending parks or converting degraded areas to community gardens, supports population health by managing urban heat islands, air pollution and flood risk. The enhancement of urban aquatic environments, such as restoration of wetlands and retention ponds for stormwater run-off, contributes to flood control and water conservation. Both strategies can promote biodiversity and support greenhouse gas retention.^14^^,^^23^ These nature-based solutions can also contribute to improved land and water management (SDGs 14 and 15) and reduced hunger (SDG 2). Involvement of city health departments can help to ensure that these adaptation actions target the most vulnerable people and can provide greater awareness of the broad range of health benefits towards behaviour change. Yet research suggests that public health departments have been among the least engaged in city adaptation planning and that the benefits to health from greenhouse gas emissions reductions (often called health co-benefits) are often overlooked.^24^^,^^25^

Another way in which cities are responding is by sharing experiences via knowledge networks. The Milan Urban Food Policy Pact aims to develop sustainable urban food systems,^26^ and monitors city activities through six indicator categories developed by FAO (food governance, equity, diet and nutrition, food supply, distribution and waste).^27^ The EAT–Cities network has published food policy recommendations, including zoning for urban agriculture, strategies for reducing food transport emissions, and upgrading sewage treatment to protect aquatic systems.^28^ Such networks build on the longer-term efforts of alliances towards sustainable agriculture. Two key examples are the Resilient Cities initiative of the Consortium of International Agricultural Research Centers, which supports urban and peri-urban agriculture and food in low- and middle-income countries;^29^ and the partnership between the Convention on Biological Diversity and Local Governments for Sustainability, aimed at enhancing city awareness and policy action on biodiversity.^30^ These initiatives are targeted at reducing both over- and undernutrition, lowering greenhouse gas emissions, improving people’s livelihoods and achieving multiple SDGs.

However, few systematic assessments of adaptation actions to address agricultural biodiversity and its relationship with health have been published. A review of national adaptation plans in 50 lower-income countries found that few plans incorporated health in a comprehensive way.^31^ A systematic review found limited examples of urban agriculture and biodiversity, mainly in North America, and pointed to the need for more research to demonstrate the impact on well-being.^32^ An FAO review of the activities of the Milan Urban Food Policy Pact identified promising results of food-related initiatives, but found that most cities had weak food and nutrition governance which was often integrated into other sectors such as public health, water and sanitation or land use.^33^ To address this gap in the literature, I aimed to identify the scope and nature of agricultural biodiversity actions within the climate adaptation plans of a sample of large world cities. I also evaluated the extent of health engagement (health sector involvement or health co-benefits) in these actions and assessed differences across country income groups.

## Methods

### Data source

For this descriptive study I evaluated data from the 2021 Cities Climate Adaptation Actions database curated by the Carbon Disclosure Project. This organization hosts a suite of databases forming the unified reporting framework for a consortium of city climate change networks, including: Local Governments for Sustainability, the Global Covenant of Mayors, C40 Cities and others.^34^ The Climate Adaptation Actions database is the most extensive and consistent publicly available data set on self-reported actions on climate change adaptation by world cities. The database is updated annually by authorized local government officials via an online questionnaire that employs a menu of categories in response to a set of standard questions. Responses are reviewed and validated by staff of the database curator who also provide guidance to cities in completing the questionnaires.^35^ In the 2021 edition of the database, 573 cities completed questionnaires on their climate adaptation actions. All actions reported to the 2021 database were actions that cities were committed to, either in preparation or under some form of implementation during that year. 

### Data collection

I aimed to focus on a city sample with a sizeable population vulnerable to climate hazards, biodiversity loss and significant greenhouse gas emissions. I therefore chose large cities as the focus for this study, selecting cities reporting to the 2021 database if the population exceeded 1 million and if a description of at least one adaptation action was provided. For cities meeting these criteria, I downloaded each city’s descriptions of their climate adaptation actions, their implementation progress, and the climate hazards their city faces from the Carbon Disclosure Project Cities, States and Regions Open Data Portal on 12 September 2022. I also extracted cities’ responses to survey questions regarding the sectors that the adaptation action applies to (for which Public Health and Safety is one menu option) and co-benefit areas (for which Improved Public Health is one menu option). I collated these data on an Excel spreadsheet (Microsoft Corp., Redmond, United States of America).

### Data analysis

The structure for the analysis was an urban agricultural biodiversity framework which I adapted from another study.^17^ The definition of agricultural biodiversity, or agrobiodiversity, I adopted was “the variety and variability of animals, plants and micro-organisms that are used directly or indirectly for food and agriculture… as well as the diversity of the agro-ecosystems.”^36^ The categories proposed in the framework align with the EAT-*Lancet* strategic goals and provide a structure for considering city activities and health impacts. As adapted for my study, the framework consisted of five categories: (i) urban and peri-urban land use; (ii) urban and peri-urban water management; (iii) urban food supply chains; (iv) urban food availability; and (v) urban food environments.^17^ More details are in the author’s online repository.^37^ Cities with programmes spanning categories may have more robust agrobiodiversity, and more meaningful impact on planetary and human health.

To conduct the analysis, I reviewed all descriptions of climate adaptation actions reported by the selected cities (using translations for non-English language descriptions) and identified actions involving agriculture (including forestry, aquaculture or horticulture), and ecosystems or biodiversity. For each action, I determined which category it fitted in the urban agrobiodiversity framework. In some cases, reported actions contained more than one different intervention or policy and I therefore separated these and eliminated any duplicate actions. I subsequently re-checked the analysis for any errors or misclassifications. I then identified and aggregated, and subsequently re-checked, the reported health and safety co-benefits; health and safety sector involvement; and implementation status for each city. I also extracted and analysed the means of implementing the actions and associated climate hazards. Finally, as context, I extracted data on climate-related health issues and vulnerable groups reported by a subset of these large cities to the 2020 Carbon Disclosure Project Cities Adaptation Actions database. 

The main study results are shown here as number and share of cities reporting at least one adaptation action that came under one of the framework categories. Also shown are number and share of cities with at least one action with health sector involvement or health co-benefits. To examine differences across cities, I stratified the results by World Bank country income category,^38^ comparing all cities in low- and middle-income countries with those in high-income countries.

## Results

### Cities and health context

A total of 141 large world cities reporting 973 adaptation actions met the population and data inclusion criteria of the study. Of these, 94 (67%) of cities were located in low- and middle-income countries (mainly in Africa, Latin America, South and South-East Asia) and 47 (33%) in high-income countries (mainly in Europe and North America). The climate hazards most frequently being addressed were extreme heat (92 cities; 65% of the total), followed by flooding (90 cities; 64%), extreme precipitation (58 cities; 41%) and drought (42 cities; 30%). 

A subset of 70 cities reported climate-related health issues; the sample was similarly distributed across low-, middle- and high-income countries as the main sample. These cities most frequently expressed current concern for air pollution-related illness (46 cities; 66%), vector-borne disease (45 cities; 64%) and heat-related illness (40 cities; 57%). Concern for noncommunicable diseases (32 cities; 46%) and food- and waterborne diseases (22 cities; 31%) were also reported, while nutrition outcomes and mental illness were among the least reported health concerns (15 cities; 21%, and 13 cities; 19%, respectively). 

Among 72 cities reporting climate-vulnerable populations, elderly people and children were most frequently reported (63 cities; 88%, and 52 cities; 72%, respectively), followed by people with low incomes (44 cities; 61%) and those living in poor-quality housing (40 cities; 56%).

### Urban agrobiodiversity

A total of 61 of the 141 cities (43%) reported 142 adaptive actions that fitted within the urban agrobiodiversity and health framework. Every world region was represented, although Brazil, Canada, Colombia, Italy, Malaysia, Mexico, Peru, Philippines, Türkiye, the United Kingdom of Great Britain and Northern Ireland, and the United States of America each had two or more cities represented (more details in the online repository).^37^

Of 142 agrobiodiversity actions, 118 actions (83%) were under implementation, operational or being monitored, while 23 actions (17%) were in the earlier stages (scoping or pre-feasibility studies of actions). The largest single means of implementation was through infrastructure actions (55 actions; 39%), while other important types of action were awareness raising, policy and regulation, stakeholder engagement and capacity-building.

Among the 61 cities with agrobiodiversity actions, urban and peri-urban land use was the category most frequently reported (39 cities; 64%), followed by urban and peri-urban water management (26 cities; 43%; Table 1). Fewer cities reported actions in the categories of urban food supply chains (six cities; 10%), food choices (15 cities; 25%) or food environment (14 cities; 23%). Forty cities reported health co-benefits or health-sector involvement in at least one of their agrobiodiversity actions. Involvement in the public health sector was reported by 27 cities (44%) and health co-benefits were reported by 31 cities (51%). For actions on urban and peri-urban land use, 21 cities (34%) reported health co-benefits (over half of cities reporting that category) and 13 cities (21%) reported health-sector involvement (one third of cities reporting that category); 10 cities (16%) reported both health co-benefits and health sector involvement for water management activities (nearly 40% of cities reporting that activity). The share of cities reporting health engagement was lower for the other categories.

**Table 1:** Agrobiodiversity action plans in 61 large cities, by category and country income group, 2021

Urban agrobiodiversity, categories and actions^a^	No. of cities reporting agrobiodiversity actions (%)		
	Low- and middle-income countries (*n* = 43)	High-income countries (*n* = 18)	Total (*n* = 61)
**Urban and peri-urban land use**			
Land use management, soil protection to minimize climate risks; support peri-urban agriculture, greenhouse gas absorption	5 (12)	1 (6)	6 (10)
Development of climate-resilient crops, trees; seed banking	1 (2)	1 (6)	2 (3)
Public parks, urban greening, nature-based solutions including for heat management, air pollution reduction, biodiversity protection	26 (60)	10 (56)	36 (59)
Eradication of invasive species threatening food crops or promoting vector-borne disease	1 (2)	1 (6)	2 (3)
Total^b^	28 (65)	11 (61)	39 (64)
**Urban and peri-urban water management**			
Coastal, watershed or riverbank land use management; wetland restoration to protect native and remove alien species, greenhouse gas absorption	11 (26)	2 (11)	13 (21)
Flood management through blue nature-based solutions	4 (9)	5 (28)	9 (15)
Water conservation, reuse, quality management for agriculture	7 (16)	1 (6)	8 (13)
Total^b^	20 (47)	6 (33)	26 (43)
**Urban food supply chains**			
Initiatives to better link peri-urban agriculture with urban centre, including infrastructure and risk reduction	1 (2)	2 (11)	3 (5)
Early warning and preparedness, mapping of crop vulnerability from harms due to climate hazards of flood and drought	1 (2)	0 (0)	1 (2)
Building of agriculture business skills to account for need for climate adaptation	2 (5)	0 (0)	2 (3)
Total^b^	4 (9)	2 (11)	6 (10)
**Urban food choices**			
Support for city and peri-urban community agriculture, aquaculture and horticulture, including organic farming	7 (16)	2 (11)	9 (15)
Provision of food supplies in extreme weather emergencies, food safety monitoring	2 (5)	0 (0)	2 (3)
City food and nutrition awareness campaigns and policies, including food sovereignty, reduced meat and others	7 (16)	2 (11)	9 (15)
Total^b^	12 (28)	3 (17)	15 (25)
**Urban food environment**			
Promotion of traditional local agricultural knowledge	2 (5)	0 (0)	2 (3)
Green buildings, including with edible food component; fish farming basins	3 (7)	6 (33)	9 (15)
Food waste reuse, composting	4 (9)	1 (6)	5 (8)
Total^b^	8 (19)	6 (33)	14 (23)

^a^ The actions reported by cities were fitted into categories in a framework^37^ adapted from a previous urban agricultural biodiversity framework.^17^

^b^ Totals are for any action in the category. *n* is the total number of cities in each income group. Figures for subcategories do not add to category totals as cities may have reported actions in multiple subcategories. Percentages are rounded.

Note: Cities reporting to the 2021 Cities Climate Adaptation Actions database of the Carbon Disclosure Project^34^ were included if the population exceeded 1 million and if a description of at least one adaptation action was provided.

Twelve cities reported four or more agrobiodiversity actions. Of these, eight cities reported actions across at least four of the urban agrobiodiversity categories: Bogotá, Colombia; Curitiba, Brazil; Denizli, Türkiye; Lima, Peru; Milan, Italy; Quezon City, Philippines; Paris, France; and Surat, India (Box 1).

Box 1 **Examples of cities’ agricultural biodiversity actions**

**Urban and peri-urban land use**

In Denizli (Türkiye), the peri-urban erosion reduction programme maps flood-related erosion risk, identifies afforestation priorities and supports farmers to improve terracing, pasturing and wildfire risk identification. Among other actions are a forest management programme in Amman (Jordan) and financing for private sector investment in urban and peri-urban tree planting in Manchester (United Kingdom of Great Britain and Northern Ireland).

**Urban and peri-urban water management**

The restoration programme for mangrove forests in Seberang Perai (Malaysia) supports the fishing industry and enhances carbon retention. Other actions are programmes for water reuse, water-efficient irrigation and drought-resistant crops in Casablanca (Morocco) and the beach recovery plan in Barcelona (Spain) that addresses conservation and the effects of warming seawater on fish and marine species.

**Urban food-chains**

In Toronto (Canada), an examination of the city’s food supply chain used an assessment of the risks from climate-related extreme weather to develop peri-urban farming alternatives. Other initiatives include an agricultural drought early warning programme in Izmir (Türkiye) and a training programme for agriculture companies and farmers on the business implications of climate resilience in Ekurhuleni (South Africa).

**Urban food choices**

Urban agriculture and community garden programmes in Curitiba (Brazil) make use of abandoned spaces for organic farming, renewable energy, reuse of materials and greenhouse gas absorption. Paris (France) funds organic farming on city reservoirs via its water company, while Lima (Peru) reports a campaign on awareness of healthy food and Cali (Colombia) has launched a formal food independence policy.

**Urban food environment**

Milan (Italy) has an urban greening programme that provides for biodiversity, greenhouse gas retention, water conservation, heat management and nutrition benefits. Kaohsiung (China, Taiwan) reported a pilot study of a roof garden, while Quezon City (Philippines) provides food for humanitarian shelters in the context of climate-related extreme flooding, and has enhanced food safety monitoring for periods of extreme heat.

Source: The examples were extracted from the 2021 Cities Climate Adaptation Actions database curated by the Carbon Disclosure Project

### Differences across income

**Fig. 1:**
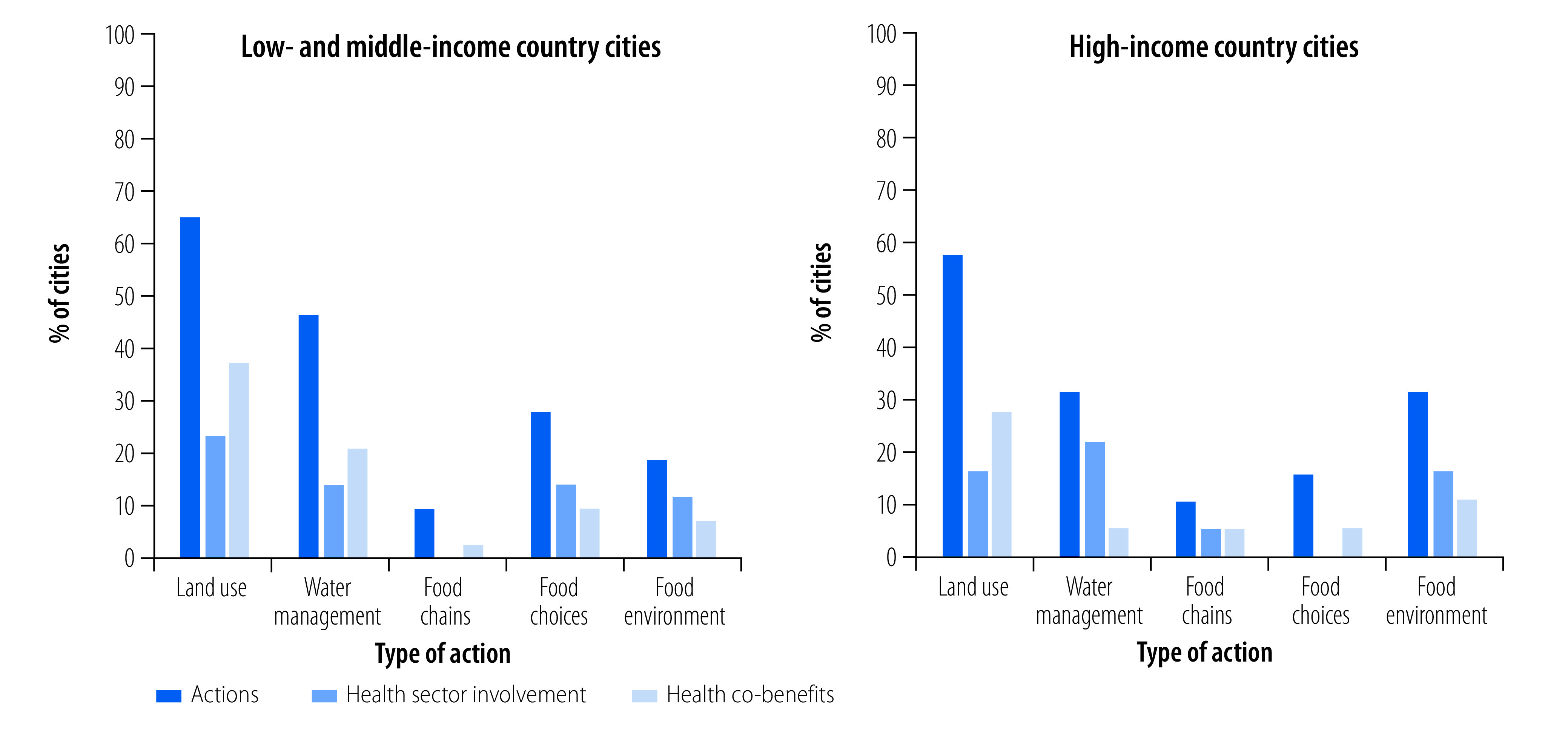
Agrobiodiversity actions, health sector involvement and health co-benefits in 61 large cities, by country income group, 2021

Note: Cities reporting to the 2021 Cities Climate Adaptation Actions database of the Carbon Disclosure Project^34^ were included if the population exceeded 1 million and if a description of at least one adaptation action was provided. A total of 43 of cities were located in low- and middle-income countries and 18 in high-income countries. The actions reported by cities were fitted into categories in a framework^37^ adapted from a previous urban agricultural biodiversity framework.^17^ Income groups are those of the World Bank.^38^

A higher share of the cities in low- and middle-income countries (43/94 cities; 46%) reported any urban agrobiodiversity action compared with cities in high-income countries (18/47 cities; 38%). The agrobiodiversity actions were also more likely to have health sector involvement or health co-benefits in cities in low- and middle-income countries (29/43 cities; 67%) than cities in high-income countries (11/18 cities; 61%; Fig. 1). Health co-benefits were recognized by a larger share of cities in low- and middle-income countries (24 cities; 56%) than high-income countries (7 cities; 39%). However, high-income cities were more likely to report health sector involvement (9 cities; 50%) than were low-income cities (17 cities; 42%). Low- and middle-income country cities reported a higher share of co-benefits in land use, water management and food choices. 

Most of the cities with more comprehensive agrobiodiversity programmes were in low- and middle-income countries (Box 1). These cities were also more likely to report health sector involvement (though not health co-benefits) than the rest of the sample. Common strategies used were food or nutrition awareness campaigns or policies, urban or peri-urban agriculture programmes, and community gardens, which were reported by a higher share of these cities than in the rest of the sample.

## Discussion

This study found evidence of modest but important action to enhance urban agrobiodiversity in the sample of large world cities reviewed. Urban and peri-urban land use and water management were the predominant categories reported, providing potential health benefits including reductions in air pollution-, heat- and vector-related illness, and flood impacts. Notably, these benefits largely align with the principal reported health concerns of the administrations of these large cities. Conversely, actions on food supply chains, food choices and food environment were less commonly reported. Of note, improved nutrition, a health benefit of food-related interventions (whether in response to insufficient or excess nutrients) was rarely reported by cities as a health issue of concern. Given the relative newness of city-based food or agriculture and biodiversity policies, these mixed results are not surprising.^14^^,^^33^ The low priority placed on nutrition outcomes and actions may warrant further research and policy focus. The lack of reported action on food-chains is also concerning, given the impact of urban food transport on greenhouse gas emissions.

Just under half of cities reported health sector involvement in agrobiodiversity actions (higher for food choices and food environment) and over half reported health co-benefits (higher for peri-urban land and water management). Health therefore played a greater role in cities’ agrobiodiversity activity than might be expected based on previous research.^24^^,^^25^ This finding adds to more recent literature identifying the health sector as a key partner for urban climate actions.^39^^,^^40^ Placing a greater emphasis on health is an actionable and scalable approach to greater sustainability in urban agrobiodiversity. Such an approach can, for example, help with targeting of vulnerable populations and monitoring of population well-being in priority areas such as nutrition.^9^

The impact of support from past and current international programmes and collaborative initiatives was evident from this analysis. Some of the strongest urban agrobiodiversity performers were low- and middle-income country cities which have benefited from FAO and other related programmes. Examples are Lima in Peru and Quezon City and Davao in the Philippines, which have been Resilient Cities target countries,^29^ and Quito in Ecuador, which has been an early pilot city in the Milan Urban Food Policy Pact network.^33^ Research examining data from 80 countries to develop an agrobiodiversity index rated just 12 countries with high scores.^41^ Four of these countries were present in the current study, each with at least one large city reporting agrobiodiversity actions: Brazil (eight cities), France (one city), South Africa and the United Kingdom (two cities each). Similarly, several cities identified in this study as reporting multiple agrobiodiversity actions have taken the initiative to report results to the Singapore City Biodiversity Index (including Barcelona, Spain; Bogotá, Colombia; Cape Town, South Africa; London, United Kingdom; and Toronto, Canada).^42^

This study provides a systematic comparison of urban agrobiodiversity actions based on the most comprehensive available international database of cities’ climate adaptation actions. However, there are several limitations. The Carbon Disclosure Project database reports actions being implemented by a sample of participating network member cities and is not a representative sample of large world cities. While the database’s validation procedures reduce the risk of error in self-reported responses, other shortcomings exist, including different interpretations of the meaning of action across cities. For this reason, my study relied on the share of cities reporting any action. Lack of survey questions regarding agrobiodiversity may also have hindered responses (on food-chains, for example). Over time, further refinements to the database may be helpful in addressing this issue. In addition, some sources suggest that urban agrobiodiversity activity, particularly food-related action, is more extensive than reflected here. For example, more comprehensive food and nutrition programmes are reported for Barcelona, which has hosted the Milan Urban Food Policy Pact network.^43^ Finally, while this study focused on large cities, medium and small cities are often closely linked to the rural economy and therefore play a key role in transforming the food system towards greater health and sustainability.^44^ Not including medium and small cities in this study does not diminish their importance. Thus, the study findings should be seen as an indicative rather than a representative line of evidence.

The findings suggest that there are opportunities for promoting policies in support of healthier, more sustainable food and biodiversity programmes through city climate adaptation plans. One opportunity is building on health sector engagement in city adaptation plans to further enhance the interconnections between agriculture and biodiversity. Research suggests that when the public health sector is involved, climate adaptation outcomes may be better targeted towards those in greatest need.^45^^,^^46^ Viewing agrobiodiversity through the lens of health can increase knowledge and awareness in the population and provide compelling reasons for behaviour change. Including health-linked urban agrobiodiversity indicators within climate adaptation plans may be especially relevant.^27^ Such indicators, which city public health agencies often track, include undernutrition (prevalence of child stunting, wasting, access to safe clean water), excess nutrition (prevalence of overweight and obesity, diabetes type 2), and equity (percentage of food-insecure households). In the present study, city administrators reported low concern for nutrition outcomes and fewer food-related adaptation actions. These results suggest that the health perspective could be harnessed for greater focus on both under- and overnutrition in city climate adaptation plans. Other related priorities include greater awareness of factors such as the health benefits of reducing air pollution with lower greenhouse gas emissions; reducing contact with wild animals or infectious diseases by the restoration of animal habitats; and reducing economic and social vulnerability by improving housing quality.

A second opportunity is to integrate agrobiodiversity goals more fully into current and future nature-based solutions. Existing efforts may already be contributing to the goals of urban agrobiodiversity, even if the descriptions of cities’ adaptation actions do not show this. Current approaches may be readily modifiable, such as by incorporating urban agriculture;^47^ training urban farmers to diversify to native species; or testing trials of water-resistant crops.^48^ Finally, the cross-sector partnerships of city adaptation plans – which often involve urban planning, public health, emergency management, utilities, weather services, and others^45^ – may help avoid some potential for unintended consequences with individual agrobiodiversity actions.^48^^,^^49^

The findings presented here underline that cities are key partners in building a more sustainable global agriculture system that addresses the twin challenges of climate change and biodiversity loss. The results also suggest that city climate action plans with a strong emphasis on health present a promising opportunity to strengthen policy and practice focus on agrobiodiversity towards more sustainable human and planetary health.
